# Rifampicin-Manuka Honey Combinations Are Superior to Other Antibiotic-Manuka Honey Combinations in Eradicating *Staphylococcus aureus* Biofilms

**DOI:** 10.3389/fmicb.2017.02653

**Published:** 2018-01-11

**Authors:** Michael Y. Liu, Nural N. Cokcetin, Jing Lu, Lynne Turnbull, Dee A. Carter, Cynthia B. Whitchurch, Elizabeth J. Harry

**Affiliations:** ^1^The ithree Institute, University of Technology Sydney, Ultimo, NSW, Australia; ^2^School of Life and Environmental Sciences, University of Sydney, Sydney, NSW, Australia

**Keywords:** medihoney, *Staphylococcus aureus*, manuka, antibiotic, synergy, biofilm, MacSynergy II, natural product

## Abstract

Chronic wound infections are a major burden to both society and the health care industry. Bacterial biofilms are the major cause of chronic wound infections and are notoriously recalcitrant to treatments with antibiotics, making them difficult to eradicate. Thus, new approaches are required to combat biofilms in chronic wounds. One possible approach is to use drug combination therapies. Manuka honey has potent broad-spectrum antibacterial activity and has previously shown synergistic activity in combination with antibiotics against common wound pathogens, including *Staphylococcus aureus*. In addition, manuka honey exhibits anti-biofilm activity, thereby warranting the investigation of its potential as a combination therapy with antibiotics for the topical treatment of biofilm-related infections. Here we report the first use of MacSynergy II to investigate the response of established *S. aureus* (strain NCTC 8325) biofilms to treatment by combinations of Medihoney (medical grade manuka honey) and conventional antibiotics that are used for preventing or treating infections: rifampicin, oxacillin, fusidic acid, clindamycin, and gentamicin. Using checkerboard microdilution assays, viability assays and MacSynergy II analysis we show that the Medihoney-rifampicin combination was more effective than combinations using the other antibiotics against established staphylococcal biofilms. Medihoney and rifampicin were strongly synergistic in their ability to reduce both biofilm biomass and the viability of embedded *S. aureus* cells at a level that is likely to be significant *in vivo*. Other combinations of Medihoney and antibiotic produced an interesting array of effects: Medihoney-fusidic acid treatment showed minor synergistic activity, and Medihoney-clindamycin, -gentamicin, and -oxacillin combinations showed overall antagonistic effects when the honey was used at sub-inhibitory concentration, due to enhanced biofilm formation at these concentrations which could not be counteracted by the antibiotics. However, these combinations were not antagonistic when honey was used at the inhibitory concentration. Confocal scanning laser microscopy confirmed that different honey-antibiotic combination treatments could eradicate biofilms. Our results suggest that honey has potential as an adjunct treatment with rifampicin for chronic wounds infected with staphylococcal biofilms. We also show that MacSynergy II allows a comprehensive examination of the synergistic effects of honey-antibiotic combinations, and can help to identify doses for clinical use.

## Introduction

The incidence, cost, morbidity, and mortality associated with chronic wound infections are a major burden to both society and the health care industry. In Australia alone, an estimated 400,000 people suffer from chronic wounds or ulcers at an estimated cost of AU$2.8 billion annually and ~AU$10, 000 per patient per year (Graves and Zheng, 2014; Whitlock et al., 2014). In the United States, chronic wounds affect 6.5 million people and cost US$25 billion yearly (Sen et al., 2009). Although bacteria living in both planktonic (free-living) and biofilm states can cause infections in wounds, biofilms cause most chronic wound infections, leading to significant delays in wound healing (Percival et al., 2012; Zhao et al., 2013). Biofilms are up to 1,000 times more recalcitrant to treatment with antibiotics and biocides than planktonic cells (Hoyle and Costerton, 1991; Hall-Stoodley and Stoodley, 2009; Lebeaux et al., 2014), making them difficult to eradicate using antibiotic therapy.

One possible approach to eradicate bacterial biofilms is to use drug combination therapy, which can widen the therapeutic window, lower the dosage, and reduce the development of antimicrobial resistance (Bjarnsholt et al., 2013; Wu et al., 2015). Honey, a natural product that exhibits potent broad-spectrum antibacterial (bactericidal) activity (Carter et al., 2016) has shown synergistic activity in combination therapy with certain antibiotics for planktonic cells (Jenkins and Cooper, 2012b; Müller et al., 2013; Liu et al., 2015).

Manuka honey, derived from nectar collected by honey bees (*Apis mellifera*) foraging on *Leptospermum scoparium* plants, is the predominant antimicrobial honey in clinical use. Key to manuka honey is the reactive dicarbonyl methylglyoxal (MGO) (Adams et al., 2008; Mavric et al., 2008) which, together with high sugar content, low water activity, low pH, and the formation of hydrogen peroxide (H_2_O_2_) upon dilution, produces potent antibacterial activity (Allen et al., 1991; Bogdanov et al., 2008). Honey also has a low propensity for bacterial resistance (Blair et al., 2009; Cooper et al., 2010), a highly attractive property given the global antibiotic resistance crisis. The need for alternative strategies to treat infections, particularly by reducing or avoiding the use of our most potent antibiotics for superficial infections, has never been greater. Honey dressings and wound gels, licensed by regulatory authorities in many countries, are currently available for clinical use. However, honey remains under-utilized as a wound treatment as health professionals claim more experimental data are required to support clinical use (Cooper and Jenkins, 2012; Carter et al., 2016). A recent Cochrane review concluded that there is currently insufficient robust evidence to determine the value of honey as a treatment for chronic wounds, although it notes that honey does heal partial thickness burns and infected post-operative wounds more quickly than conventional treatments (Jull et al., 2015).

*Staphylococcus aureus* is a common cause of chronic wound infections (Bowler et al., 2001). We and others have demonstrated strong synergy between manuka honey and rifampicin, oxacillin, and clindamycin in inhibiting the growth of planktonic cells and preventing biofilm formation by different *S. aureus* strains [including methicillin-resistant *S. aureus* (MRSA)] (Jenkins and Cooper, 2012a,b; Müller et al., 2013; Liu et al., 2015). In some cases, antibiotic-resistant strains became markedly more sensitive to antibiotics in the presence of manuka honey (Müller et al., 2013; Lu et al., 2014; Liu et al., 2015). However, infected chronic wounds typically have established biofilms, and reducing or eliminating these is of clinical importance. Manuka honey alone can reduce established *S. aureus* biofilms (Lu et al., 2014) but this has not yet been tested in combination with antibiotics. This study therefore set out to investigate interactions between honey and antibiotics on established biofilms.

Establishing drug interactions can be challenging and there are various models available (Greco et al., 1995). To date, interaction studies using honey have employed the fractional inhibitory concentration index (FICI) (Jenkins and Cooper, 2012a,b; Müller et al., 2013; Liu et al., 2015). However, this has disadvantages when assessing honey-antibiotic interactions because (1) the minimum inhibitory concentration (MIC) for honey is relatively high compared to antibiotics and expressed using different units, i.e., percentage weight per volume (% w/v) as opposed to μg/ml; (2) synergy by FICI requires a dose reduction in both agents, and honey-antibiotic combinations often lead to a marked reduction in the dose required of antibiotic but not honey (Liu et al., 2015); and (3) FICI is a two-dimensional (2-D) analysis whereby one variable must be constant and as such, it does not allow comprehensive dose-response analyses, potentially missing or underestimating important interactions (Greco et al., 1995). To overcome these limitations, models have been developed that enable three variables (concentrations of both drugs and the biological effect) to be analyzed at once. Plotting the dose-response in a three-dimensional (3-D) space allows the identification of both synergistic and antagonistic interactions across a range of concentrations for both drugs.

Here we evaluate the response of established *S. aureus* NCTC 8325 biofilms to treatment by combinations of Medihoney and conventional antibiotics frequently used systemically to treat infected chronic wounds: rifampicin, oxacillin, fusidic acid, clindamycin, and gentamicin, using MacSynergy II to construct a 3-D drug interaction surface (Prichard et al., 1991). We show that the Medihoney-rifampicin combination was most effective combination at eradicating established staphylococcal biofilms. The results of this study suggest that honey could be used in combination therapy with rifampicin for chronic wounds with established biofilms caused by *Staphylococcus* (including MRSA), adding weight to the synergistic anti-staphylococcal activity of this combination treatment previously reported against planktonic cells and biofilm prevention (Müller et al., 2013; Liu et al., 2015). We also show that MacSynergy II allows identification of doses with synergistic activity that are otherwise overlooked in conventional 2-D analyses (FICI).

## Materials and methods

### Bacterial strain, antibiotics, and honey

Laboratory reference strain *S. aureus* NCTC 8325 was used in this study as it has the ability to form robust biofilms on abiotic surfaces (Lu et al., 2014) and was used in our earlier studies of biofilm and planktonic cell responses to Medihoney and antibiotics (Müller et al., 2013; Liu et al., 2015). Tryptone soya broth (TSB, Oxoid) was used for all growth assays and biofilm treatment assays. Antibiotics (rifampicin, fusidic acid, clindamycin hydrochloride, gentamicin sulfate solution and oxacillin sodium salt) were purchased from Sigma-Aldrich. Medihoney, a commercially available medical-grade honey (predominantly derived from *L. scoparium* blended with some *Kunzea ericoides* honey), was provided by Comvita Ltd, New Zealand. The honey was stored in the dark at 4°C and was freshly diluted for use in every assay. The concentrations of the two major antimicrobial components (MGO, H_2_O_2_) were tested and were equivalent to previously reported levels (MGO: 776 mg/kg; H_2_O_2_: 0.31 μmol/h) (Lu et al., 2013, 2014). Honey concentrations are reported in this study as % weight per volume (w/v).

### Determination of MICs for antimicrobial agents and their ability to reduce established biofilms

The MICs of Medihoney and antibiotics against planktonic *S. aureus* were determined via broth microdilution assays, as previously described (Liu et al., 2015). The MIC was assessed as the lowest concentration that inhibited 99% of *S. aureus* compared to the untreated (growth) control.

The ability of antimicrobial agents to reduce established *S. aureus* biofilms was assessed using a biofilm treatment assay as previously described (Lu et al., 2014). Briefly, biofilms were established in each well of a sterile 96-well flat-bottomed microtiter-plate by inoculating approximately 10^7^ CFU/mL of *S. aureus* in TSB and incubating at 37°C for 24 h without shaking. Biofilms were then rinsed twice with sterile phosphate buffered saline (PBS) and treated with either Medihoney (concentrations ranging from 1–32%, equivalent to 0.125–4 × MIC, in two-fold increments) or antibiotics (at concentrations that were 1, 3, 5, 10, 30, 50, and 100 × MIC) and incubated for a further 24 h at 37°C. Following incubation, the liquid content from each well was carefully removed with a pipette and the wells washed twice by gently rinsing with PBS to remove loosely attached planktonic cells. The remaining biofilms were dry-fixed onto the plates by air-drying at 65°C for 1 h and stained with 0.2% crystal violet solution for 1 h at room temperature. Excess crystal violet solution was decanted and the plates rinsed with sterile reverse osmosis (RO) water and briefly dried before solubilising the stained biofilm with 30% acetic acid. The OD (595 nm) of each well was measured on an automated microtiter plate reader (VersaMax, Molecular Devices, California, USA) and the readings were converted into percentage of biofilm reduction based on the untreated control wells where no antibiotic or Medihoney was applied.

### Checkerboard microdilution assay to assess medihoney-antibiotic interactions against *S. aureus* biofilms

*S. aureus* biofilms established as above were washed with sterile PBS and each pair of antimicrobial agents (Medihoney and antibiotic) was added across the x and y dimensions of the 96-well plate in two-fold serial dilutions with TSB medium. Concentrations tested ranged from 0.25–4 × MIC for each antibiotic and from 0.125–8 × MIC (i.e., 1–32% w/v) Medihoney. Each combination was repeated in the adjacent horizontal wells to provide technical duplicates, and the experiment was performed on 3 different days to provide experimental replicates. The experiment included two treatment controls, where either antibiotic or Medihoney was used alone, and a negative control (media only).

### Statistical analysis of honey-antibiotic interactions using MacSynergy II

Dose-response curves were plotted to determine if a honey-antibiotic combination pair reduced the biofilm biomass more than either honey or antibiotic used alone. MacSynergy II (Prichard et al., 1991) is based on a Bliss Independence null reference model and uses the dose response curves of the individual antimicrobials (honey or antibiotic) to calculate theoretical additive interactions and to produce a predicted additive surface, with the assumption that the agents act independently (Prichard and Shipman, 1990). An interaction is defined as synergistic if the observed surface is greater than the predicted additive surface when confidence intervals are set at 95%. Antagonism is defined as the observed surface being less than the predicted additive surface.

### Viability assay using bactiter-glo

The viability of biofilms remaining post-treatment was evaluated using the BacTiter-Glo^TM^ (Promega, Madison WI) assay, which measures the production of adenosine triphosphate (ATP) by metabolically active bacterial cells using a luminescent reporter. As ATP production is relatively constant across many different growth conditions (Schneider and Gourse, 2004), ATP-based assays are used routinely to monitor the presence of active bacteria and have been applied to biofilms (Takahashi et al., 1988; Monzón et al., 2001; Romanova et al., 2007; Sule et al., 2009; Lu et al., 2014).

After honey and antibiotic treatment of established biofilms as outlined above, BacTiter-Glo reagent (20 μl) was added into each well followed by 100 μl of TSB. The bioluminescence reaction was started by the addition of the BacTiter-Glo reagent and incubation in the dark for 10 min at 37°C. Bioluminescence was determined in a Tecan Infinite 200 PRO series microplate reader (Tecan Group, Switzerland). To allow for consistency in incubation times, a single 96-well microtiter-plate was processed at a time. Statistical analysis to determine significant differences in viability following treatments was performed using one-way ANOVA with the Tukey test in GraphPad Prism (version 6; CA, USA) with statistical significance set at *p* <0.05.

### Visual assessment of biofilm reduction using confocal microscopy

For confocal microscopy, treatments were set up as described above, but in black polystyrene 96-well microtiter plates (μClear® bottom Cellstar®; Greiner Bio-One, France). Following treatment, the plate was washed three times with PBS and cells within the biofilm structure were fluorescently stained with 2.5 μM SYTO^TM^ 9 Stain (Invitrogen, CA, USA) and 4.3 μM propidium iodine (PI) (Becton Dickinson, NJ, USA), which identify live and dead cells, respectively, in the biofilm structure. After 30 min of incubation in the dark at room temperature, the wells were washed with PBS and fixed with 4% paraformaldehyde (Sigma-Aldrich, MO, USA) for 15 min. The wells were then rinsed and stored in PBS for imaging. Biofilms were imaged using confocal laser scanning microscopy (CLSM) imaging on a Nikon A1 confocal microscope (with 40X Plan Fluor ELWD, 0.6 N/A objective). The SYTO® 9 and PI fluorophores were excited at 488 and 561 nm, and the emissions were collected at 500–550 nm and 570–620 nm, respectively. Representative image stacks of each treatment were acquired at a resolution of 1024 × 1024 pixels and biofilm image reconstructions were performed using NIS-elements (version 10, Nikon Instruments Inc., USA).

## Results

### Minimum inhibitory concentrations and anti-biofilm activity of medihoney and antibiotics against *S. aureus*

We used broth microdilution growth assays to determine the individual MICs of Medihoney and selected antibiotics against planktonic growth of *S. aureus* NCTC 8325 (Table 1). Consistent with our previous studies (Lu et al., 2013, 2014; Müller et al., 2013; Liu et al., 2015), the MIC of Medihoney was 8% w/v. The MICs for the selected antibiotics against *S. aureus* are comparable to those reported in the literature (O'Neill et al., 2002; Hanssen et al., 2004; Didier et al., 2011).

**Table 1:** Summary of the results of Medihoney and antibiotic treatments on *S. aureus*.

			**Checkerboard combination assay with Medihoney**				
			**Microdilution assay**^c^		**MacSynergy II analysis**^d^		**Effect on cell viability^e^**
	**MIC^a^ (% w/v or μg/ml)**	**Biofilm eradication^b^ (% w/v or μg/ml)**	**Additive interaction**	**Honey-antibiotic concentrations with additive interaction (% w/v–μg/ml)**	**Overall MacSynergy conclusion**	**Honey-antibiotic concentrations with significant synergistic peaks (% w/v–μg/ml)**	
Medihoney	8	16	N/A	N/A	N/A	N/A	N/A
Rifampicin	0.04	>4	Yes	8–0.01 8–0.02	Strongly synergistic	1–0.01 2–0.04 4–0.01 4–0.04 8–0.01	Significant
Clindamycin	0.08	0.8	Yes	8–0.02 8–0.04	Antagonistic	2–0.04 2–0.32	Significant
Fusidic acid	0.08	2.4	Yes	8–0.02 8–0.04	Synergistic, minor	8–0.02 8–0.04 8–0.16	Not significant
Gentamicin	0.626	>62.5	No	N/A	Antagonistic	N/A	N/A
Oxacillin	0.16	0.48	No	N/A	Antagonistic	N/A	N/A

a *MIC: minimum inhibitory concentration, expressed as the mean % weight per volume for honey or μg/ml for antibiotics from three separate experiments*.

b *Data presented in Figure 1. Results expressed as concentration of agent required to achieve ≥85% eradication compared to the untreated control, % weight per volume for honey or μg/ml for antibiotics*.

c *Data presented in Figure 2, concentrations expressed as % weight per volume for honey and μg/ml for antibiotic*.

d *Data presented in Figure 3, concentrations expressed as % weight per volume for honey and μg/ml for antibiotic*.

e *Data presented in Figure 4, left panel. Statistical significance determined using one-way ANOVA, p < 0.05*.

*N/A, not applicable*.

The individual effect of Medihoney and selected antibiotics on the biomass of established *S. aureus* biofilms was determined using a biofilm treatment assay based on crystal violet staining (Table 1). The established biofilm decreased in biomass when treated with Medihoney at 1–4 × MIC or each of the five selected antibiotics at 1–100 × MIC. The extent of biofilm reduction differed for the different agents. When honey was used at the MIC (8% w/v), biofilm biomass was reduced by 25%. Thus, at the MIC honey is less effective than rifampicin, fusidic acid, clindamycin, and oxacillin at reducing biofilm biomass (reduced by 65, 55, 65, and 80%, respectively), but more effective than gentamicin (reduced by 15%). However, at 2 × MIC (16% w/v) honey was effective at reducing the established biofilm by 90%. Of the antibiotics, oxacillin was the most effective giving ~90% reduction when used at 3 × MIC and up to 95% reduction when used at 5 × MIC. Clindamycin and fusidic acid required 30 × MIC for a reduction of ~90%, while gentamicin and rifampicin did not reduce the biofilm to this level at any concentration tested, and plateaued at ~80% reduction at 50 × MIC. Complete biofilm eradication (i.e., 100% reduction) was not observed for any of the antibacterial agents, even at the highest concentrations tested (4 × MIC for honey; 100 × MIC for antibiotics; Figure 1); however a reduction of biofilm by ≥85% was defined as eradication as consistent with other biofilm studies.

**Figure 1:**
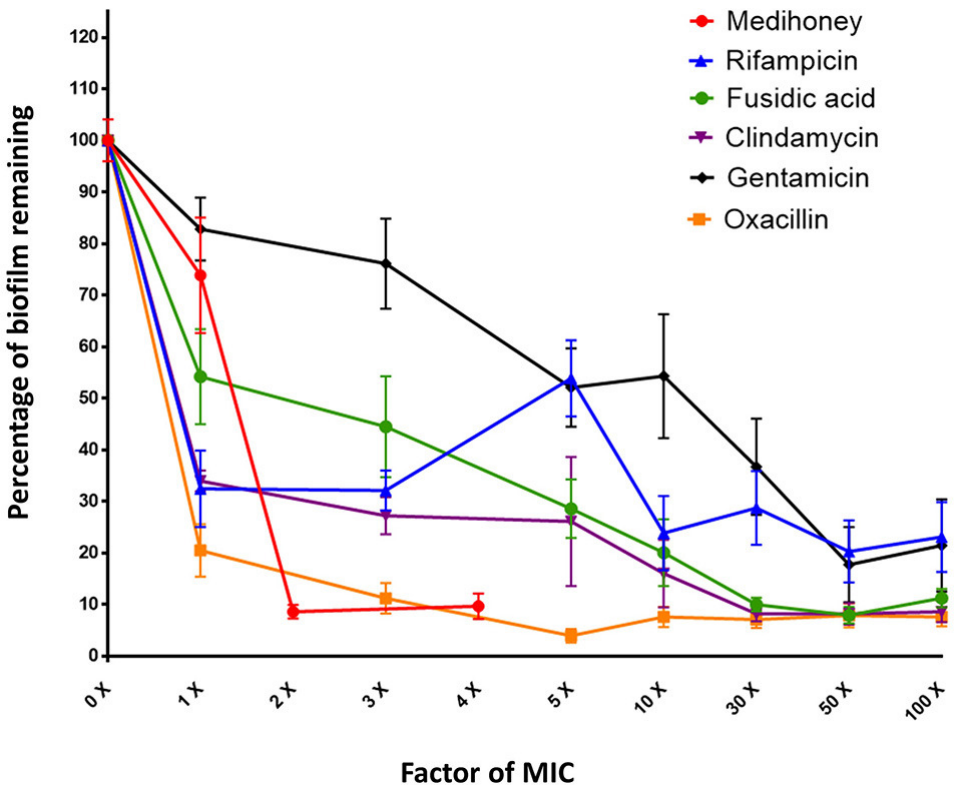
Dose response curves for established *S. aureus* biofilms treated with Medihoney and antibiotics. Biofilms were established during 24 h static incubation before treatment for 24 h with either Medihoney or antibiotic at a range of concentrations. Biofilm biomass was then quantified using crystal violet staining. The biofilm remaining is expressed as a percentage relative to that of the untreated control, which is set at 100%. Error bars represent the standard error of the mean (SEM) of triplicate samples performed in duplicate on 3 different days.

### Interactions between medihoney and antibiotics on established *S. aureus* biofilms

**Figure 2:**
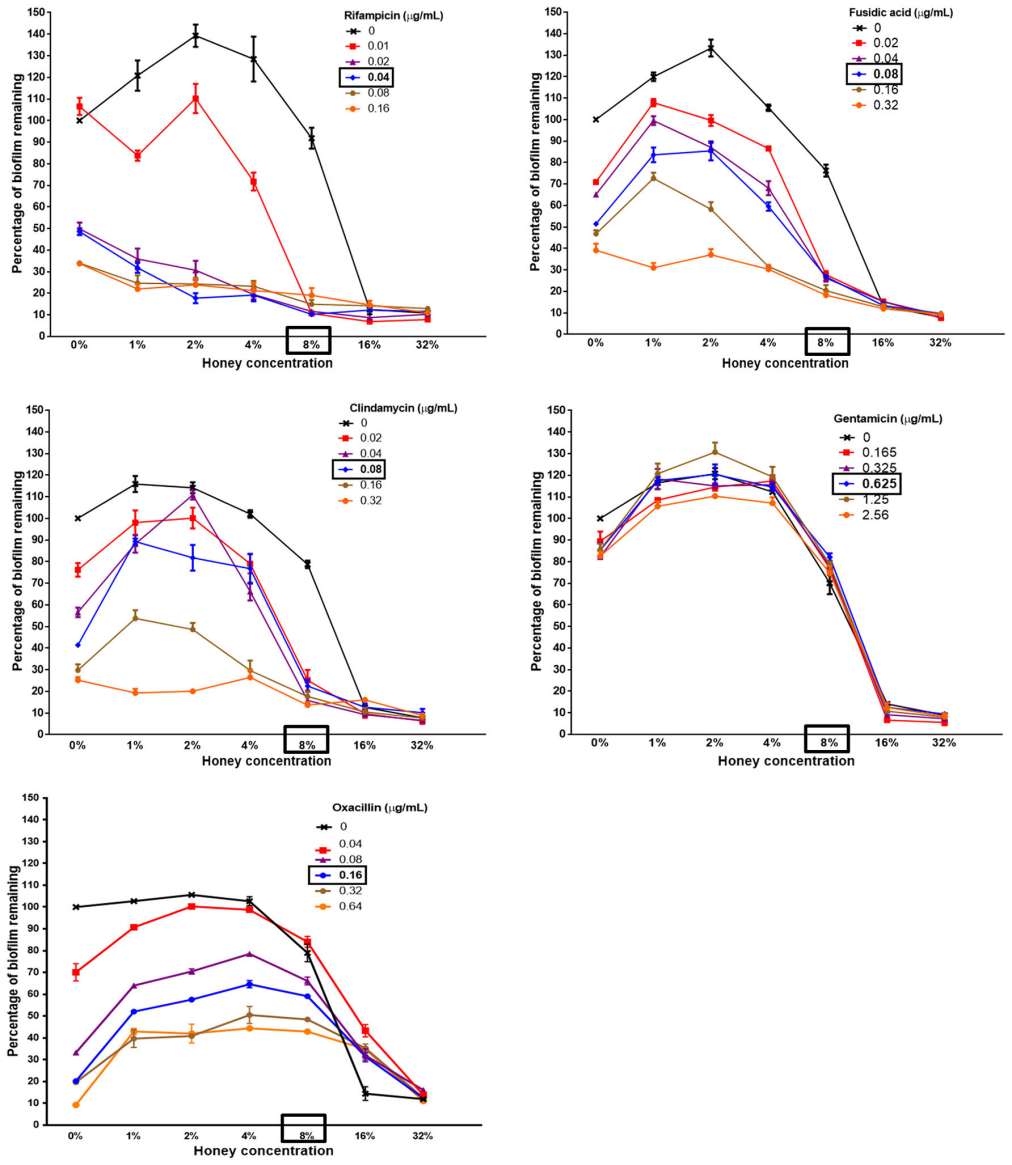
The ability of Medihoney-antibiotic combinations to remove biofilm varies greatly with different antibiotics Medihoney-rifampicin combinatorial treatment showed the greatest reduction in biofilm even when the antibiotic was used at sub-inhibitory levels. Medihoney combined with either fusidic acid or clindamycin also reduced biofilm substantially. Combinations of Medihoney with gentamicin or oxacillin did not reduce biofilm any more than that which was achieved by the antibiotic alone. Error bars represent the standard error of the mean (SEM) of triplicate samples performed in duplicate. The MIC of each antibacterial agent is shown in bold and boxed.

We used a checkerboard microdilution assay based on crystal violet staining to determine whether the treatment of established biofilms with combinations of Medihoney and a selected antibiotic would reduce biofilm biomass more than treatment with Medihoney or antibiotic alone. In the combinatorial treatments, Medihoney was used at 0.125–4 × MIC (1–32% w/v) and each antibiotic was used at 0.25–4 × MIC (in two-fold increments), and the results are summarized as dose-response curves (Figure 2).

The established biofilms increased in biomass when Medihoney was used at concentrations below the MIC (1–4% w/v; 0.125–0.5 × MIC) and low levels of the antibiotics largely paralleled this pattern. We noted a similar trend previously when this *S. aureus* strain was treated with Medihoney (Lu et al., 2014).

The effect of Medihoney-antibiotic combinations on established biofilms varied depending on the antibiotic used and on the concentrations of the agents (i.e., honey and antibiotic; Figure 2 and Table 1). The most striking positive effect was observed for the Medihoney-rifampicin combination, where biofilm biomass reduction was much larger than what was achieved by Medihoney or rifampicin used alone. This was observed across multiple concentration combinations of Medihoney-rifampicin, including concentrations below the MIC. For example, 8% w/v Medihoney (MIC) on its own reduced biofilm biomass by < 10% but this increased to 85% when combined with sub-inhibitory rifampicin concentrations (0.02 μg/ml; 0.5 × MIC). Sub-inhibitory concentrations of Medihoney (2 and 4% w/v; 0.25–0.5 × MIC) and rifampicin (≥0.02 μg/ml; 0.5 × MIC) when used together reduced biofilm biomass by at least 70%. For both the Medihoney-clindamycin and Medihoney-fusidic acid combinations, substantial biofilm reduction (> 70%) was observed when the Medihoney was used at the MIC (8%) and antibiotics used at any concentration, including sub-inhibitory ones (0.02–0.04 μg/ml; 0.25 0 0.5 × MIC). No significant reduction in biofilm biomass was observed for either the Medihoney-oxacillin or the Medihoney-gentamicin combinations compared to that achieved by the antibiotic alone.

### MacSynergy II plots reveal the landscape of synergy and antagonism in medihoney-antibiotic combinations on established biofilms

**Figure 3:**
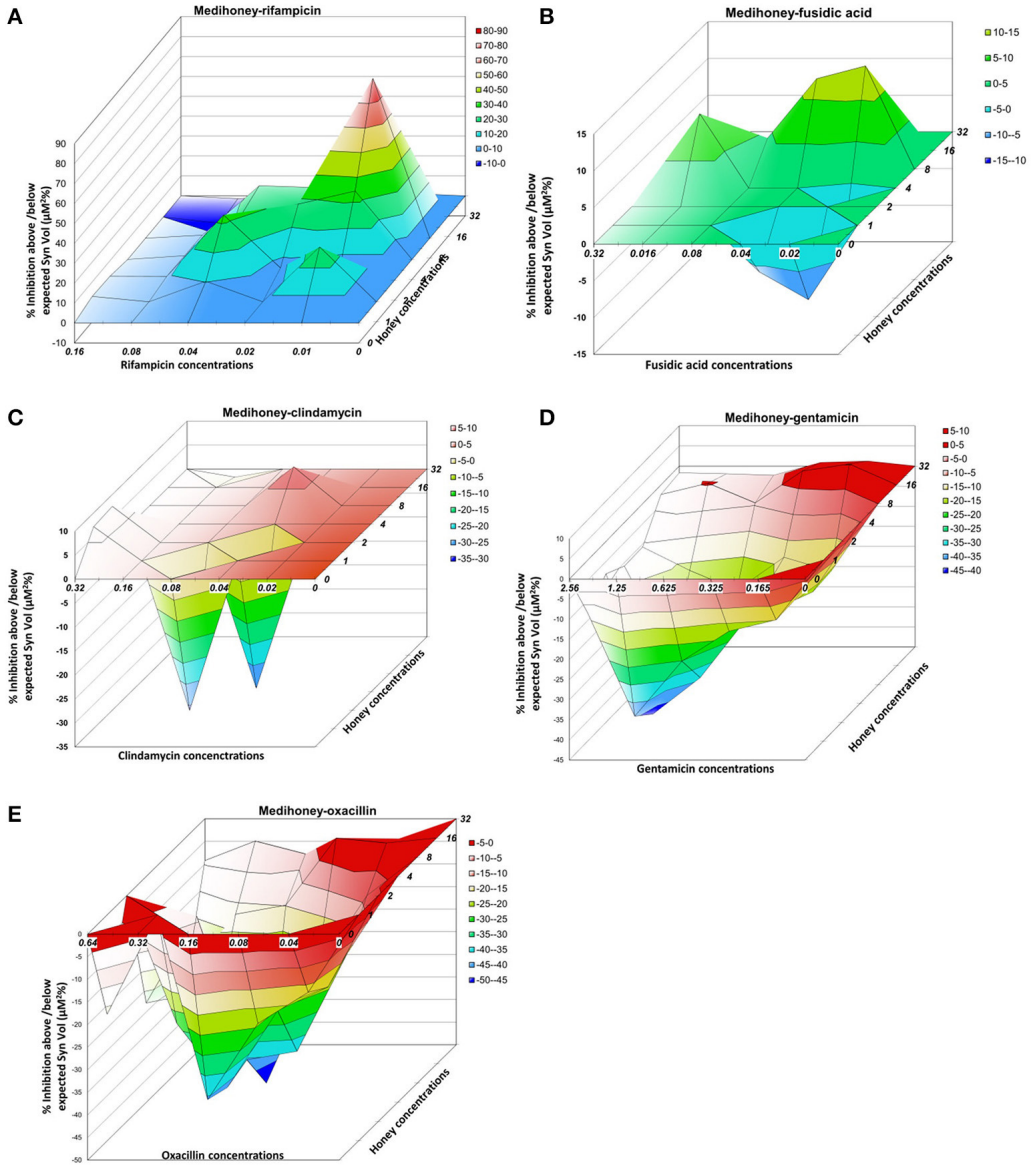
Three-dimensional dose-response plots highlight substantial differences in the effect of Medihoney-antibiotic combinations on established *S. aureus* biofilms Interaction surfaces were calculated based on response surface analysis using MacSynergy II. Additive interactions appear as a horizontal plane at 0% inhibition. Peaks of statistically significant synergy (positive value) or antagonism (negative value, troughs) that deviate significantly from the theoretical expected additive interaction surface are shown, with different colors indicating the level of synergy or antagonism. Antibiotic concentrations (x-axis) are in μg/ml and honey (z-axis) in % weight per volume. Plots show combinations of Medihoney with: **(A)** rifampicin; **(B)** fusidic acid; **(C)** clindamycin; **(D)** gentamicin; and **(E)** oxacillin.

To better visualize the nature and the degree of interactions that occur between Medihoney and the selected antibiotics in the treatment of established biofilms, we performed a rigorous quantitative analysis of their interaction using MacSynergy II (Prichard and Shipman, 1990). The program uses the spectrometric absorbance data generated by the checkerboard dilution assays and plots a landscape of the dose-response, rather than a curve, enabling a visual representation of the combined doses where the response is synergistic (peaks), additive (horizontal plane) or antagonistic (trough; Figure 3, Table 1).

For the Medihoney-rifampicin combinations, all interactions were above the plane of additivity (Figure 3A), and the overall synergy/antagonism volumes were 305/−6 μM^2^% (Supplementary Table 1). Thus, the observed size of the synergy derived from the 95% confidence interval (based on Bonferroni adjustment) indicated a strong synergistic interaction between Medihoney and rifampicin across a range of concentrations (volumes >50 denote synergy). Moreover, this is probably important *in vivo* (volumes >100 identified as having likely significance *in vivo* Prichard and Shipman, 1990). The Medihoney-fusidic acid combination produced a complex interaction profile consistent with the curves presented in Figure 2, with synergy (peaks) at high concentrations of both agents and antagonism (troughs) at low concentrations (Figure 3B). The observed synergy volumes seen when the higher concentrations of Medihoney-fusidic acid were used corresponded to a volume of 42.49 μM^2^% (Supplementary Table 1), considered significant but minor (Prichard and Shipman, 1990). The antagonism trough (volume −27.24 μM^2^%) observed with 1% Medihoney−0.02 μg/ml fusidic acid (equivalent to 0.125 and 0.25 × MIC), respectively, was not significant.

The Medihoney-clindamycin combination showed a small peak of synergy (Figure 3C; volume 23 μM^2^%, Supplementary Table 1) and a more pronounced antagonism volume of −63 μM^2^%, identifying concentrations that are potentially useful (i.e., synergistic) and those that should be avoided (i.e., antagonistic). Finally, MacSynergy analysis of the Medihoney-gentamicin and Medihoney-oxacillin combinations demonstrate strong antagonistic effects for almost all concentration combinations (Figures 3D,E) with overall antagonism volumes of −422.17 and −1278.96 μM^2^%, respectively (Supplementary Table 1). This is in agreement with the dose-response curves where no positive interactions were detected by these combined treatments on established biofilms (Figure 2).

### Effect of medihoney and antibiotic combinations on biofilm cell viability and biofilm structure

Although the checkerboard microdilution assay using crystal violet staining revealed certain Medihoney and antibiotic combinations resulted in significant changes to established biofilm biomass, this assay does not assess the viability of cells remaining within the biofilm structure (Bauer et al., 2013). To quantitatively assess biofilm cell viability we used the BacTiter-Glo assay, which measures ATP levels as a proxy for viability. We have previously correlated the luminescent signal from the ATP levels to *S. aureus* cell numbers (CFU/ml) via direct enumeration (Lu et al., 2014). The effect of the treatment combinations on established biofilms at the cellular level was analyzed using confocal scanning laser microscopy with the “live” cell stain, SYTO^TM^ 9. We performed these experiments using treatments with honey combined with rifampicin, clindamycin, or fusidic acid since these antibiotics showed some synergy with honey in the dose-response curves and MacSynergy assays (Figures 2, 3).

**Figure 4:**
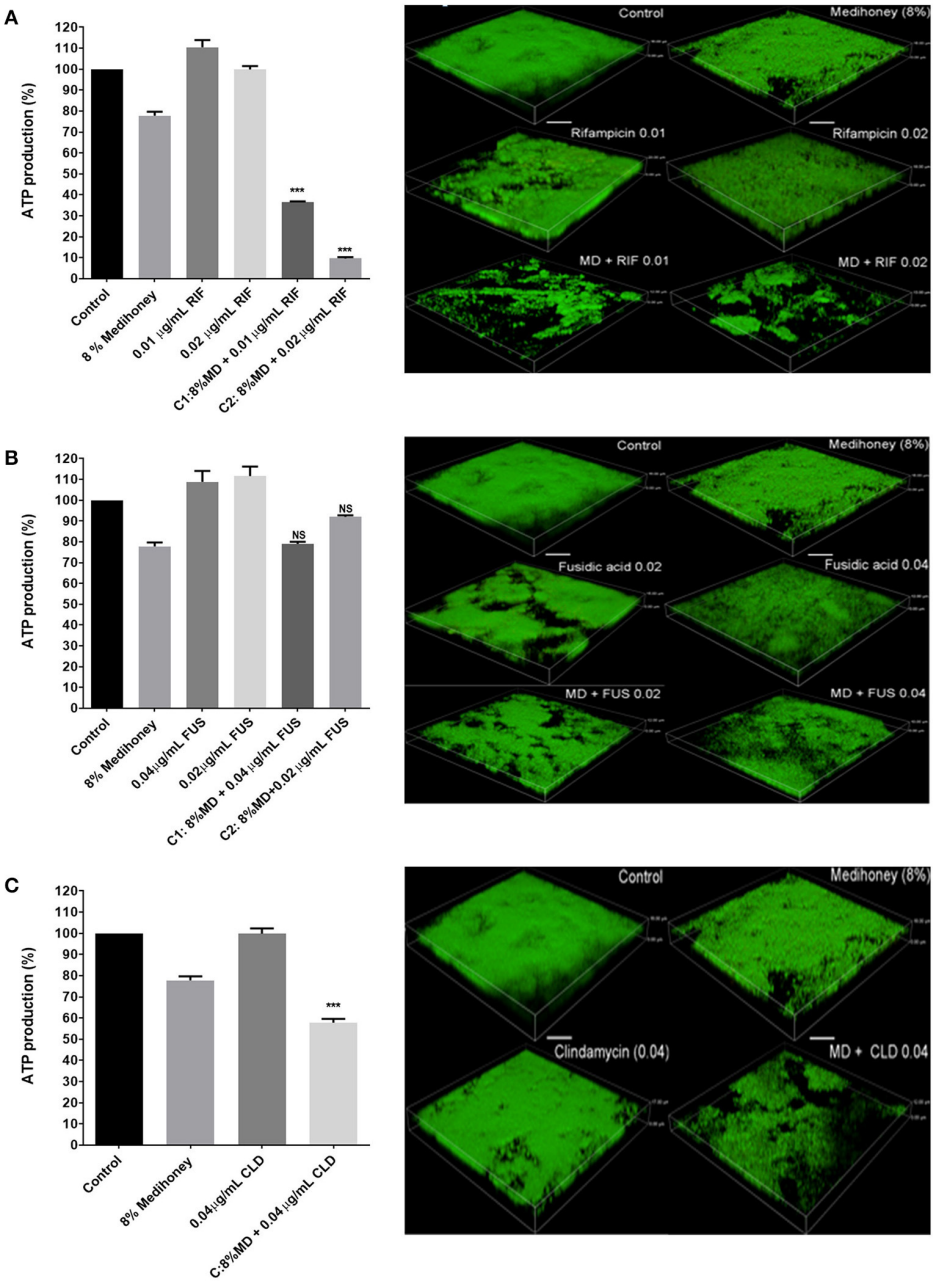
Effects of Medihoney and antibiotic combinations on cell viability within biofilms and on biofilm structure. Medihoney (MD) was tested in combination with each of three antibiotics: **(A)** Rifampicin (RIF); **(B)** Fusidic Acid (FUS); **(C)** Clindamycin (CLD). Left panels: viable cells remaining within the established biofilm following treatment with Medihoney-antibiotic combinations were evaluated using the BacTiter-Glo assay, which measures the production of ATP. The percentage of viable cells was calculated relative to the untreated control, which was set at 100%. Statistical significance (*p* < 0.05) was assessed by one-way ANOVA with a Tukey post-hoc test. Medihoney-fusidic acid was the only tested combination found to be not significant (NS). Right panel: 3-D images produced by confocal laser scanning microscopy of established biofilm after combination treatment. Biofilms were stained with SYTO^TM^ 9 (green = viable cells). Both the thickness and structure of the established biofilms were notably reduced after treatment by Medihoney-rifampicin and Medihoney-clindamycin. Scale bar represents 10 μm.

Cells within the biofilm that remained following treatment with synergistic concentrations of Medihoney-rifampicin had a significant reduction in viability compared to cells within untreated biofilms. For example, 8% w/v Medihoney + 0.01 μg/ml rifampicin gave > 60% reduction in viability; and 8% w/v Medihoney + 0.02 μg/ml rifampicin gave a 90% reduction (*p* <0.005, Figure 4A, left panel). Similarly, a reduction in density of cells was observed using confocal laser scanning microscopy (Figure 4A, right panel). The Medihoney-rifampicin combination thus demonstrated strong synergistic effects in both reducing biofilm biomass and in reducing the viability and density of cells within the biofilm that remained following treatment. These results are consistent with the checkerboard analysis.

Despite minor synergism in reducing biofilm biomass observed when Medihoney (8% w/v) was combined with 0.02 μg/ml or 0.04 μg/ml fusidic acid (Figure 2), the cells in the remaining biofilm did not show reduced viability compared to the single-treatment controls (i.e., cells in biofilms treated with the same concentrations of Medihoney or fusidic acid alone). Similarly, this combination did not produce a significant decrease in biofilm live-cell density relative to the controls (Figure 4B, right panel). This suggests that these treatment combinations may be effective in reducing biofilm biomass but are less effective as a bactericidal against cells in an established biofilm.

In contrast, Medihoney-clindamycin, which was overall antagonistic in its effects on biofilm biomass (Figures 2, 3), did significantly reduce the viability of cells in the remaining biofilm when compared to cells within untreated biofilms or biofilms treated with clindamycin or Medihoney alone (Figure 4C, left panel; *p* < 0.05). Further, confocal microscopy showed a marked reduction in density of the remaining biofilm (Figure 4C, right panel).

## Discussion

The incidence of non-healing chronic wounds within the community is increasing largely due to rising rates of diabetes and obesity, and the increase in the aging population (Sen et al., 2009). Chronic wounds harbor bacterial populations that commonly exist as biofilms (Dowd et al., 2008). This makes chronic wounds inherently difficult to treat with antibiotics because bacteria present in biofilms are far more tolerant of antibiotics than planktonic bacteria (Percival et al., 2012; Zhao et al., 2013). Therefore, effective treatments of chronic wounds need to be able to reduce the existing biofilms in the wound and simultaneously prevent the formation of new biofilms.

Honey-antibiotic treatments of *S. aureus* cells have shown promise in previous studies, whereby the inhibition of planktonic bacterial cells and prevention of biofilms was improved when honey was used in combination with certain antibiotics (Müller et al., 2013; Lu et al., 2014; Liu et al., 2015). Here we show, for the first time, the effects of honey-antibiotic combinations on *in vitro* established biofilms using MacSynergy II to plot the entire dose-response landscape. We hypothesized that a combination of Medihoney, a clinically available honey in a proprietary formulation, and conventional antibiotics would be synergistic in their effect on established *S. aureus* biofilms. We found, however, that a variety of responses to the Medihoney-antibiotic combinations occurs, with some being antagonistic, depending on the antibiotic and concentrations used.

Standing out from this complexity was the Medihoney-rifampicin combination on established biofilms. Rifampicin alone did not have very high potency against biofilms, but was drastically altered in the presence of honey showing strong synergistic activity across all honey and drug concentrations (Figures 1, 2). Analysis of the checkerboard data for biofilm biomass reduction using MacSynergy II indicated this synergy was strong enough to expect it to be significant *in vivo* (Figure 3, Table 1). Accordingly, cell viability was significantly reduced and there was a marked reduction in the live-cell biofilm density (Figure 4). These findings support our previous reports of synergism between Medihoney and rifampicin against *S. aureus* in planktonic and biofilm form (Müller et al., 2013; Liu et al., 2015). Rifampicin as a treatment on its own has been associated with the rapid emergence of resistance in *S. aureus* (Raad et al., 2007), but has been identified as an anti-staphylococcal antibiotic enhancer in combination therapies with vancomycin, tetracycline, gentamicin, fusidic acid, oxacillin, ciprofloxacin, and cefazolin (Monzón et al., 2001; Saginur et al., 2006; Raad et al., 2007). For example, a rifampicin-vancomycin combination was found to have increased efficacy against *S. aureus* biofilms, purportedly because the drugs had complementary killing activity whereby vancomycin was more effective at eradicating early stage biofilms and rifampicin on mature biofilms (Monzón et al., 2001). One proposed reason for rifampicin acting as an enhancer in anti-biofilm drug combinations has been that rifampicin acts to decrease adherence of biofilm organisms to surfaces, causing the release of increasing numbers of bacteria off the biofilm and into broth, therefore allowing the antibiotics to diffuse and kill the bacterial cells (Monzón et al., 2001; Saginur et al., 2006). Additionally, it has been proposed that the bactericidal, rather than bacteriostatic, activity of rifampicin aids in synergistic activity (Monzón et al., 2001). Our findings also give weight to these studies, identifying rifampicin as a superior candidate for combinational approaches in preventing and eradicating staphylococcal biofilms. The use of *in vivo* approaches, such as wound or animal models, to demonstrate the synergistic effects of the Medihoney-rifampicin combination would further support the prediction power of the *in vitro* studies performed here.

For the other Medihoney-antibiotic combinations, the results were very different. Some Medihoney-antibiotic combinations were antagonistic (gentamicin and oxacillin), and some (fusidic acid clindamycin) had both synergistic and antagonistic interactions depending on the concentrations used (Figure 3). Antagonism seems to be largely driven by the biofilm-enhancing action of honey when used at sub-MIC doses combined with the ability of the antibiotic to counteract this effect. For example, gentamicin cannot counteract this biofilm-enhancing action and all concentrations of it largely parallel the honey response (Figure 2). Clindamycin, fusidic acid, and oxacillin have an intermediate ability to reduce the biofilm-enhancing action, and this increases with increased concentrations of antibiotic. The biofilm-enhancing action of low doses (<MIC) of honey has been reported previously (Lu et al., 2013), and could be due to a stress response, which has been observed when bacteria in biofilms are exposed to sub-inhibitory concentrations of antibiotics (Haddadin et al., 2010; Subrt et al., 2011; Kaplan et al., 2012). It should be noted that the biofilm-enhancing action of sub-inhibitory honey concentrations is not due to the extra sugar available to the organism as sugar solution controls were not found to enhance biofilm (Lu et al., 2013).

Previous studies have investigated the effect of Medihoney-antibiotic combinations on *S. aureus*, both in the planktonic state and on biofilm formation (Liu et al., 2015). The type of effect—as determined by FICI analysis of Medihoney-antibiotic combinations—was found to be identical for planktonic growth and biofilm formation and no antagonism was observed for any combination studied. Synergy was observed between Medihoney and each of oxacillin, clindamycin and rifampicin. The Medihoney-gentamicin combination however showed no synergistic activity, nor did it show antagonism (fusidic acid was not tested). This unity of effect contrasts with the complex effects observed in the current study using established *S. aureus* biofilms (Table 1), however one point of clear consistency is the strong synergy produced by Medihoney-rifampicin combinations against *S. aureus*, whether planktonic or in biofilms.

This is, to our knowledge, the first study to examine the honey-antibiotic interactions using MacSynergy II. By simultaneously visualizing antagonistic and synergistic areas in the same plot, MacSynergy II makes it possible to find a combined drug dose that shows synergy which can be overlooked when using a single value, such as the FICI. This was particularly useful because of the observation that sub-inhibitory levels of Medihoney or antibiotics actually enhance rather than reduce biofilms, therefore analyses that rely on generating one overall synergy score (e.g., FICI) may distort our ability to detect synergy when it does happen. For example, fusidic acid and clindamycin, which are of intermediate potency when used alone, showed improved anti-biofilm activity when combined with Medihoney, generally following a dose-dependent manner (Figures 2, 3). MacSynergy II analyses are therefore useful for informing the correct doses to use in order to enhance the anti-biofilm activity of these inhibitors.

The importance of assessing cell viability and visually inspecting biofilms is best demonstrated by the effects of the Medihoney-clindamycin treatment. In this example, an overall antagonistic effect was assigned when the biofilm reduction data were analyzed with MacSynergy II (Figure 3, Table 1). However, confocal microscopy revealed a visible reduction in biofilm cover and a significant reduction in viability of cells in the remaining biofilm following treatment with 8% w/v Medihoney and 0.04 μg/ml clindamycin (Figure 4C). Additionally, treatment with Medihoney-fusidic acid at concentration pairs showing synergistic activity in reducing biofilm biomass (8% honey−0.02 μg/ml drug; 8% honey−0.04 μg/ml drug) still left viable cells in the remaining biofilm (Figure 4B). Therefore, visualizing the biofilms and assessing cell viability also play an integral part in identifying synergistic combinations.

Our results using established biofilms do not show the same synergy patterns as seen with planktonic cells or biofilm prevention (Liu et al., 2015). This is likely due to the different physiology and metabolism in the biofilms, and the different pathways controlling this specialized differentiation event. The dose-response methods used in this study are not able to identify the potential mechanism for Medihoney-antibiotic combinations. It has been previously suggested that the synergistic effect of Medihoney in combination with clindamycin may be due to both honey and drug acting on sequential or orthogonal steps of the protein synthesis pathway, shutting it down more effectively (Liu et al., 2015). For example, clindamycin inhibits bacterial cell growth by targeting the 50S subunits of the ribosome and honey (manuka) alters the levels of protein synthesis components, including ribosomal proteins (Blair et al., 2009; Packer et al., 2012). However, these genomic studies with honey were performed with bacteria in the planktonic state and it is likely that the mechanism for the interaction of Medihoney and antibiotics differs when bacteria are in the biofilm state. One possible explanation for the synergistic activity of the Medihoney-rifampicin combination seen here could be that they target the same pathway (i.e., transcription- RNA polymerase). Further investigation of the mechanism underlying synergism observed in this study may inform development of Medihoney-drug combinations for treatment of established biofilms in chronic wounds. The variation in honey-antibiotic interactions we observe here could be due to the varying nature of the drugs, their ability to penetrate the biofilm, and their different modes of action. The honey-antibiotic interactions may provide insight into the mode of action of honey, i.e., honey-antibiotic combinations with synergistic activity may have a similar mode of action, and those combinations with antagonistic activity have different ones. This effect has previously been noted in interaction studies between the essential oil of *Ocimum basilicum* (basil) and antibiotics imipenem and ciprofloxacin, showing synergistic and antagonistic activity, respectively (Araújo Silva et al., 2016).

In conclusion, our results demonstrate that the Medihoney-rifampicin combination was superior to combinations using the other antibiotics against established *S. aureus* biofilms. This adds to the synergistic activity of this combination treatment across planktonic growth and biofilm prevention (Müller et al., 2013; Liu et al., 2015), thus supporting the use of honey as an adjunct treatment with rifampicin for chronic wounds. We also show that MacSynergy II provides a valuable platform for analyzing synergistic activity between honey-antibiotic combinations, and when coupled with visual inspection of the biofilms can give a comprehensive assessment of combination therapies for clinical use.

## Author contributions

EH, DC, LT, and CW: contributed to the conception and design of the work; ML, NC, JL, and LT: contributed to the acquisition, analysis and interpretation of data for the work; The paper was written by ML and NC, and critically revised by JL, EH, DC, and CW.

### Conflict of interest statement

Comvita New Zealand provided partial funding and materials for the work described in the manuscript. The authors declare that the research was conducted in the absence of any commercial or financial relationships that could be construed as a potential.

## Supplementary material

Supplementary Table 1 Analysis of honey-antibiotic interactions using MacSynergy II. Overall synergy and antagonism values determined by plotting with 95% confidence intervals, based on the Bonferroni adjustment 97% (Prichard and Shipman, 1990).
